# Tropomyosin Receptor Antagonism in Cylindromatosis (TRAC), an early phase trial of a topical tropomyosin kinase inhibitor as a treatment for inherited CYLD defective skin tumours: study protocol for a randomised controlled trial

**DOI:** 10.1186/s13063-017-1812-z

**Published:** 2017-03-07

**Authors:** Amy Cranston, Deborah D. Stocken, Elaine Stamp, David Roblin, Julia Hamlin, James Langtry, Ruth Plummer, Alan Ashworth, John Burn, Neil Rajan

**Affiliations:** 1 Newcastle Clinical Trials Unit, Newcastle University, Newcastle upon Tyne, NE2 4AE UK; 2 Institute of Health and Society, Newcastle University, Newcastle upon Tyne, NE2 4AX UK; 3 The Francis Crick Institute, London, NW1 2BE UK; 4 Ziarco Pharma Ltd, Innovation House, Ramsgate Road, Sandwich, Kent CT13 9ND UK; 5 Department of Dermatology, Royal Victoria Infirmary, Newcastle upon Tyne Hospitals NHS Foundation Trust, Newcastle upon Tyne, NE1 4LP UK; 6 Northern Institute for Cancer Research, Newcastle University, Newcastle upon Tyne, NE2 4HH UK; 7 UCSF Helen Diller Family Comprehensive Cancer Centre, San Francisco, CA 94158 USA; 8 Institute of Genetic Medicine, Newcastle University, Newcastle upon Tyne, NE1 3BZ UK

**Keywords:** CYLD defective tumours, Tropomyosin receptor kinase (TRK) inhibitor, Cylindromatosis, TRK inhibition, Pegcantratinib, *CYLD*

## Abstract

**Background:**

Patients with germline mutations in a tumour suppressor gene called *CYLD* develop multiple, disfiguring, hair follicle tumours on the head and neck. The prognosis is poor, with up to one in four mutation carriers requiring complete surgical removal of the scalp. There are no effective medical alternatives to treat this condition. Whole genome molecular profiling experiments led to the discovery of an attractive molecular target in these skin tumour cells, named tropomyosin receptor kinase (TRK), upon which these cells demonstrate an oncogenic dependency in preclinical studies. Recently, the development of an ointment containing a TRK inhibitor (pegcantratinib — previously CT327 — from Creabilis SA) allowed for the assessment of TRK inhibition in tumours from patients with inherited *CYLD* mutations.

**Methods/design:**

Tropomysin Receptor Antagonism in Cylindromatosis (TRAC) is a two-part, exploratory, early phase, single-centre trial. Cohort 1 is a phase 1b open-labelled trial, and cohort 2 is a phase 2a randomised double-blinded exploratory placebo-controlled trial.

Cohort 1 will determine the safety and acceptability of applying pegcantratinib for 4 weeks to a single tumour on a *CYLD* mutation carrier that is scheduled for a routine lesion excision (*n* = 8 patients). Cohort 2 will investigate if CYLD defective tumours respond following 12 weeks of treatment with pegcantratinib. As patients have multiple tumours, we intend to treat 10 tumours in each patient, 5 with active treatment and 5 with placebo. Patients will be allocated both active and placebo treatments to be applied randomly to tumours on the left or right side. The target is to treat 150 tumours in a maximum of 20 patients. Tumour volume will be measured at baseline and at 4 and 12 weeks. The primary outcome measure is the proportion of tumours responding to treatment by 12 weeks, based on change in tumour volume, with secondary measures based on adverse event profile, treatment compliance and acceptability, changes in tumour volume and surface area, patient quality of life and pain.

**Discussion:**

Interventions for rare genetic skin diseases are often difficult to assess in an unbiased way due to small patient numbers and the challenges of incorporating adequate controls into trial design. Here we present a single-centre, randomised, placebo-controlled trial design that leverages the multiplicity of tumours seen in an inherited skin tumour syndrome that may inform the design of other studies in similar genetic diseases.

**Trial registration:**

International Standard Randomised Controlled Trial Number Registry, ISRCTN75715723. Registered on 22 October 2014.

**Electronic supplementary material:**

The online version of this article (doi:10.1186/s13063-017-1812-z) contains supplementary material, which is available to authorized users.

## Background

Patients who inherit germline mutations in a tumour suppressor gene called *CYLD* develop multiple, disfiguring, hair follicle tumours on the head and neck. The prognosis is poor, with up to one in four mutation carriers requiring complete scalp excision typically at the age of 55 [1]. These patients also have numerous tumours on the trunk that warrant surgical excision due to pain, ulceration or necrosis. Tumours have a predilection to develop on the external ear and in the ear canal, resulting in conductive deafness. Tumours arising on genital skin result in sexual dysfunction. These patients require repeated lifelong surgery to control the tumour burden. There are no effective medical alternatives to treat this orphan disease, which is thought to affect approximately 1 in 100,000 of the UK population [2]. This trial aims to examine the potential of a repurposed topical treatment to inhibit, and possibly prevent, tumour growth.

The impact of the disfiguring appearance on the quality of life of patients with this condition and the recurrent surgical treatments further emphasise the importance of this work. Patients find surgical interventions painful and time-consuming, and may have limited function and ability to work in the weeks following surgery. This scenario, and its impact on National Health Service (NHS) resources, could be revolutionised by an ointment that *CYLD* mutation carriers could apply to tumours when they first develop. This may inhibit tumour growth and subsequently reduce the number of surgical interventions required. As these tumours often develop on the head and neck, the reduction in disfiguring tumours and surgery would have a positive effect on patient quality of life. Furthermore, the reduced referral for specialist interventions such as surgery and lasers would free up these precious resources as well as reduce the patient pathway time.

Currently, these tumours are excised by specialists in dermatology and plastic surgery, with patients requiring multiple procedures over the course of a lifetime. Patients with *CYLD* mutations have complex care needs, warranting the input of different specialists in their management. Some procedures, such as laser resurfacing, represent a costly and scarce resource. Extensive surgical procedures such as scalp excision and skin grafting may warrant a general anaesthetic and an inpatient stay.

Tropomyosin receptor kinase (TRK) was discovered as a candidate target following a search for targetable kinases in inherited CYLD defective tumours using an unbiased genetic approach. DNA and RNA expression changes in fresh, snap-frozen tumours compared to adjacent, unaffected skin were characterised. This led to two key discoveries [3]. Firstly, the genetic changes in these tumours were limited, with restriction to loss of *CYLD* being the only detectable change seen. This homogeneity was exciting, as it implied that a targetable kinase discovered on this genetic background would be seen in the majority of tumours. Secondly, we discovered overexpression of TRK selectively in the tumour cells. These tumour cells overexpressed TRKB and TRKC in almost all tumours examined. The mechanism by which loss of functional CYLD results in perturbation of TRK homeostasis is not fully understood. TRK signalling has been shown to confer a survival advantage to tumour cells by increasing resistance to apoptosis and cell proliferation [4–6]. TRK has been increasingly recognised to be an oncogenic kinase that is overexpressed in several malignancies, including leukaemia and breast cancer. We demonstrated that CYLD defective tumour primary cell culture models on three-dimensional tissue culture scaffolds were highly sensitive to nanomolar levels of TRK inhibition. Proof of principle that inhibition of a key signalling pathway can restrain skin tumour growth has been described in another inherited skin tumour condition, naevoid basal cell carcinoma syndrome. In this condition, the tumour phenotype conferred by germline mutations in a tumour suppressor gene *PTCH* is inhibited by a small molecule inhibitor targeting Hedgehog signalling. This has resulted in a reduction in tumour volume and the number of surgical procedures that patients with this condition require [7]. TRK inhibitors have so far been only available in oral formulations, but the advent of pegcantratinib has made validation in a patient-relevant model possible with minimal risk of systemic adverse effects.

There are no effective medical alternatives to treat this condition, and the TRK inhibitor pegcantratinib may represent a first-in-class agent for the management of this condition if shown to be safe and effective in this trial. Pegcantratinib uses ’low systemic exposure’ technology via a mini-pegylation approach that generates molecules with physicochemical and pharmacological properties that make them suitable for topical use. Safety data are available from six completed studies where pegcantratinib has been applied topically at concentrations of up to 0.5% w/w for up to 8 weeks in 36 healthy volunteers and 188 patients with psoriasis or eczema [8, 9]. These studies have shown that pegcantratinib is well tolerated with a low incidence of adverse events and application site reactions. Pharmacokinetic samples taken in these studies confirm the absence of pegcantratinib in the systemic circulation (assay detection level 5 ng/ml), which is consistent with animal data and supportive of the theory that absorption of pegcantratinib via the skin is low following topical application.

This single-centre, early phase trial aims to evaluate an innovative approach towards managing the multiple tumours that arise in *CYLD* mutation carriers. This approach involves the use of pegcantratinib, which targets TRK expressed by the tumour cells in these patients. This selective targeting could lead to the inhibition of tumour growth and resolution of existing tumours. The prospect of being able to inhibit TRK focally in the skin using an ointment vehicle for drug delivery, without the systemic side effects seen with other TRK inhibitors [10], is an attractive option. Positive results would support a larger, multicentre, definitive trial which, if proved effective, could lead to an improvement in quality of patient care and improved cost efficiency, which are key NHS priorities.

## Methods/design

### Aims and objectives

This is an investigator-initiated trial to determine the safety and preliminary efficacy of pegcantratinib in patients with inherited CYLD defective skin tumours.

The primary objective in cohort 1 of the trial is to determine the safety of pegcantratinib application in *CYLD* mutation carriers. The primary objective in cohort 2 of the trial is to investigate if CYLD defective tumours respond to treatment with pegcantratinib.

The secondary objective for the TRAC trial in cohort 2 is to attempt to delineate the mechanism by which pegcantratinib and TRK inhibition may exert an effect on tumour volume.

### Trial design

This is a two-part, single-centre, phase 1b/2a trial investigating the safety and response of pegcantratinib in CYLD defective tumours.

### Cohort 1 design

This is a phase 1b open-label clinical trial to determine the safety of applying pegcantratinib to a preselected single tumour in a *CYLD* mutation carrier that is scheduled for routine excision. Cohort 1 will determine the safety of targeting TRK in this patient population. A preliminary group of eight patients (who are *CYLD* mutation carriers) will receive daily doses (using a standardised spatula) of the topical TRK inhibitor pegcantratinib (0.5% w/v) for 4 weeks to a preselected single tumour once a day. The treated site will be clinically assessed for skin inflammation using a modified version of the Draize score test for signs of local site reaction. The modified Draize score [11] is described in more detail in the ‘Outcome measures’ section (see also Additional file 1) later in the paper. Lack of any unexpected trial-related adverse site reactions is indicated by a modified Draize score of 3 or below over 4 weeks of treatment. Lack of reactions in at least five of eight treated patients will allow the trial to progress to cohort 2, following recommendation from an independent Data Monitoring Committee (DMC). As the gene dosage of *CYLD* in these patients is reduced in normal skin compared to healthy controls, this safety trial will determine safety in a *CYLD* mutation carrier population before looking at the efficacy of the drug in cohort 2.

Patients with a tumour that has been scheduled for excision will be eligible for inclusion in this cohort. Each patient will be required to apply pegcantratinib ointment to the tumour only for the 4-week interval leading up to surgery following screening and enrolment into this trial. An additional Consolidated Standards Of Reporting Trials (CONSORT) diagram for cohort 1 shows this process (see Additional file 2). The treated site will be assessed by a clinician for signs of local site reaction. A patient treatment questionnaire will be used to assess the patients’ acceptability of the treatment. Patients will also be asked to complete a general quality-of-life measure, the European Quality of Life-5 Dimensions (EQ5D) scale, and a dermatology-specific quality-of-life measure, the Dermatology Life Quality Index (DLQI), before commencing treatment in order to assess the impact of multiple CYLD defective tumours on their quality of life and to assess the patients’ perceptions of the disease. These data have not been captured in this patient population previously. If the trial is successful and the treatment is developed in future multicentre clinical trials, these data would be requested as a longitudinal measure.

Tumours excised will undergo routine analysis, and some of the samples will be snap-frozen and stored. They will be used for further mechanistic studies and stored for potential future analysis. The end of cohort 1 will be the date when data are obtained on the last remaining cohort 1 patient completing 4 weeks of treatment.

### Cohort 2 design

This is a single-centre, phase 2a, within-patient and randomized-by-tumour, double-blind, placebo-controlled trial to investigate if CYLD defective tumours respond to pegcantratinib. We will need 150 tumours to complete the trial; recruitment will continue until we have the 12-week assessment for the 150 tumours. We anticipate that 15–20 patients will need to be recruited to achieve this. Patients will typically have 8 or 10 eligible tumours each.

Patients will be randomised to receive active treatment on either the left or right side of the body, with the other side receiving placebo. Four or five tumours on each side will be identified at baseline for measurements during the 12-week treatment period.

Photographs will be taken and the tumours numbered clearly; this information will be offered to the patients, who can then refer to the tumour maps. Patients will be shown how to apply treatments using disposable gloves to prevent cross-contamination and given a detailed instruction sheet for reference. Volume measurements will be taken at baseline and at weeks 4 and 12 at scheduled visits. An additional CONSORT diagram and a pictorial overview for cohort 2 show this in more detail (see Additional files 3 and 4). All tumours will be assessed for tumour volume using the Life-Viz stereoscopic imaging system by a blinded assessor [12]. Patients will record application of treatment in a patient diary. Patients will be asked if any of the selected tumours have any associated pain (type and intensity) at baseline, and this question will be repeated at 4 and 12 weeks. After 12 weeks, one tumour that is treated with the active treatment and one tumour that has been treated with placebo will be biopsied to assess histological and immunohistochemical changes.

The placebo- and pegcantratinib-treated samples will undergo gene expression profiling to delineate the mechanism by which TRK inhibition may exert an effect on tumour volume. Tumours will be assessed for gene expression changes following TRK inhibition using RNA sequencing.

The end of cohort 2 will be when the last cohort 2 patient completing a 12-week assessment brings the number of assessed tumours to 150. Three months (12 weeks) has been selected as the trial endpoint, following discussions with five affected patients. This period of treatment is in keeping with most topical treatments for skin growths, which are typically given for 1–3 months. It is expected that longer periods may have a negative effect on trial adherence.

### Trial setting and target population

The TRAC trial is a single-centre trial based in Newcastle upon Tyne, UK.

An overview discussing the tumours seen in these patients, the natural history and the distribution is relevant to the design and outcome measurement in this trial. Key points are as follows:

1. *CYLD* mutation carriers develop multiple CYLD defective tumours, typically from the onset of puberty. These tumours (principally cylindromas, spiradenomas and trichoepitheliomas) are recognised to affect the head and neck as well as the torso and pubic skin.
2. We have studied the distribution of these tumours; they can be varied in size and location. Whilst penetrance is similar in males and females, severity appears to be increased in females [1].

Adult patients will be recruited from genotyped pedigrees with known *CYLD* mutations or from those who have a clinical phenotype compatible with this diagnosis, namely multiple cylindromas, spiradenomas or trichoepitheliomas. The patients recruited in cohort 1 will be those requesting and deemed suitable for surgery for tumour removal. These tumours will typically be larger ones that are painful or causing dysfunction. The distinction from the smaller lesions that are to be recruited in cohort 2 is reflected in the respective inclusion criteria described below.

### Cohort 1 inclusion criteria

The cohort 1 inclusion criteria are as follows:

- Males and females aged 18 years and older.
- Patients from genotyped pedigrees with known *CYLD* mutations or who have a clinical phenotype compatible with this diagnosis.
- Patients who are suitable for the trial will have at least one eligible tumour.
- The eligible tumour will be scheduled for removal >4 weeks from consent.
- The eligible tumour must be no more than 3 cm in size.
- For women of childbearing age, a negative pregnancy test is required prior to study entry and on completion of trial treatment. The patient must be using an adequate contraception method, defined as established use of oral, injected or implanted hormonal methods of contraception, placement of an intrauterine device (IUD) or intrauterine system (IUS), barrier methods of contraception (condom or occlusive cap), male sterilisation or true abstinence. The patient must agree to continue using this method throughout the trial and for at least 2 weeks after stopping trial treatment.
- Sexually active men must agree to use barrier forms of contraception.
- The recruiting clinician must be confident that the patient understands the consent process and has the capacity and willingness to provide fully informed consent for participation in the trial.

### Cohort 1 exclusion criteria

The cohort 1 exclusion criteria are as follows:

- Patients aged <18 years.
- Patients without CYLD defective tumours.
- CYLD defective tumours which are ulcerated (these tumours will be managed according to standard practice of care).
- An eligible tumour that is due to be removed <4 weeks from consent.
- Women who are pregnant or lactating.
- Women of childbearing age and sexually active men who do not wish to use contraception whilst on the study.
- Patients exhibiting severe incapacity of higher function such that fully informed consent cannot be achieved (to be determined by clinical judgement).
- Use of any other topically administered treatments at the treatment site.

### Cohort 2 inclusion criteria

The cohort 2 inclusion criteria are:

- Males and females aged 18 years and older.
- For women of childbearing age, a negative pregnancy test is required prior to study entry and on completion of trial treatment. The patient must be using an adequate contraception method defined as established use of oral, injected or implanted hormonal methods of contraception, placement of an IUD or IUS, barrier methods of contraception (condom or occlusive cap), male sterilisation or true abstinence. The patient must agree to continue using this method throughout the trial and for at least 2 weeks after stopping the trial medication.
- Sexually active men must agree to use barrier forms of contraception.
- Patients from genotyped pedigrees with known *CYLD* mutations or those who have a clinical phenotype compatible with this diagnosis.
- Patients will optimally have 8–10 eligible tumours.
- Eligible tumours will be ideally less than 1 cm in diameter and no more than 2 cm in diameter at the base.
- Eligible tumours must be spaced at least 1 cm apart from other eligible tumours to avoid cross-contamination.
- The recruiting clinician must be confident that the patient understands the consent process and has the capacity and willingness to provide fully informed consent for participation in the trial.
- Patients who have completed cohort 1 without adverse reaction and after completing a minimum 2-week treatment-free washout period.

### Cohort 2 exclusion criteria

The cohort 2 exclusion criteria are:

- Patients aged <18 years.
- Patients without multiple CYLD defective tumours.
- Women who are pregnant or lactating.
- Women of childbearing age and sexually active men who do not wish to use contraception whilst on the study.
- CYLD defective tumours which are ulcerated or have recently changed (these tumours will be managed according to standard practice of care).
- Patients exhibiting severe incapacity of higher function such that fully informed consent cannot be achieved (to be determined by clinical judgement).
- Significant concurrent illness.
- Patients who developed an adverse reaction to pegcantratinib in cohort 1 (modified Draize score of 4 or above).
- Patients who have taken part in cohort 1 and not completed a minimum 2-week treatment-free washout period.
- Large tumours of >2 cm base diameter will not be eligible.
- Any tumour within 10 cm of an excision scar of a cohort 1-treated site will not be eligible.
- Use of any other topically administered treatments at the treatment site.

### Screening, recruitment and consent

*CYLD* mutation carrier patients known to the clinical genetics and dermatology department in Newcastle will be reviewed by the clinical team to verify that they meet the trial inclusion criteria. Eligibility screening logs will be completed by the investigator, or delegate, to document participants’ fulfilment of the entry criteria for all patients considered for cohort 1 and cohort 2 and subsequently included or excluded. The eligibility screening logs will record the potential patient’s initials, screening date, outcome of screening, reason patient was ineligible/declined and consent date. Screening logs will also record the number of tumours each patient has for consideration for cohort 2.

Patients will be either approached during a routine outpatient appointment or invited in writing to attend a clinical appointment to discuss the trial. Where patients are likely to be travelling long distances, we will enclose a trial invitation letter and trial information sheet with their appointment letter. This will allow them more time to consider whether they are interested in taking part. If a patient is happy to do so, it is feasible for screening, recruitment procedures and baseline visit to be completed over 24 hours. This may be necessary where patients are travelling long distances so that inconvenience to patients is minimised. Screening is also permitted to occur a maximum of 7 days prior to randomisation in order to ensure that all screening data can be collected. Female patients who are of childbearing age will be given urine-based pregnancy tests prior to being randomised into either cohort 1 or cohort 2 of this trial. In cohort 2 these tests will be repeated at subsequent visits.

The clinical team members with delegated permission will explain the trial procedures to the patients and give them information about the trial treatments in cohort 1 or cohort 2 as appropriate (depending on which part of the trial is in progress). Prior to consent, patients will also be given a trial Patient Information Sheet (PIS) to take with them and read. They will be given opportunities to ask questions and as much time as they need (minimum 24 hours) to decide whether or not they wish to take part.

Those wishing to take part will provide written informed consent by signing and dating the trial consent form, which will be witnessed and dated by a member of the research team with documented, delegated responsibility to do so. For subjects who cannot consent for themselves, an appropriate independent witness will provide written consent. Only after written informed consent has been obtained will patients undergo any further trial-specific screening (i.e. screening that cannot be done by simple review of medical notes, such as pregnancy tests) and baseline clinical assessment, including skin examination. The original signed consent form will be retained in the Investigator Site File, with a copy in the clinical notes and a copy provided to the participant. The participants will specifically consent to their general practitioner being informed of their participation in the trial, and a standard letter will be sent out. The eligibility screening log will be updated to record the patient entering the trial. The right to refuse to participate without giving reason will be respected.

Due to the small subject population, the PIS and consent form for the trial will be available only in English. Via local NHS arrangements, interpreters will be made available for all visits of patients who require them either for verbal translation or for deaf subjects wishing to take part in the trial. Consent will not be taken from any participant unless the research team are confident he/she understands the information and can give fully informed consent. The screening assessment (as per clinical practice) must be fully completed and eligibility confirmed by an investigator prior to enrolment (cohort 1) or randomisation (cohort 2).

### Intervention

The TRK inhibitor pegcantratinib is a topical investigational medicinal product (IMP) currently being developed by Creabilis Limited for the treatment of pruritus and pain in dermatological conditions. This trial is the first trial to investigate the use of pegcantratinib in patients with CYLD defective skin tumours; however, the drug has been given to 36 healthy volunteers and 188 patients in previous clinical trials [8, 9]. At the first trial visit, patients will have their tumours for drug administration identified. Details for each cohort are stated in the inclusion/exclusion criteria. Patients will then be instructed on how to administer the trial medication and given instructions to take with them for reference. A new disposable glove should be used for each separate administration and disposed of after use. The amount of drug will be measured using a provided spatula. The patient will also be instructed to wash the area with warm water before administration. The patient will cover the full tumour evenly, being careful to not apply treatment to any surrounding areas. The ointment will be applied in the evening, 2 hours before bedtime.

In cohort 1 active trial medication containing pegcantratinib at 0.5% w/w will be provided as ointment in 20-g glass jars. Patients will be provided with a spatula to help them to apply the correct amount of ointment. The dose will be one application (two standardised spatulas) in the evening to each tumour as directed at the first visit by the research nurse/doctor. Application will be recorded in a patient diary. In cohort 1 the participants will treat their selected tumour with the active treatment for a 4-week period. Additional file 5 shows the full schedule of events (Standard Protocol Items: Recommendations for Interventional Trials (SPIRIT) figure) for cohort 1.

In cohort 2 active and placebo trial medication will be provided at baseline and at the week 4 visit to supply enough ointment for the 12-week period. Application will be as outlined for cohort 1 but one dose will be one standardised spatula per tumour. Patients will record application of treatment in a patient diary. In cohort 2 all participants will be treating tumours on one half of the body with active pegcantratinib and on the other half of the body with placebo according to the randomisation allocation for a 12-week period. A 3-day visit window is allowed for the second dispensing visit at week 4. The full schedule of events (SPIRIT figure) for cohort 2 is shown in Additional file 6.

A telephone contact number will also be provided so participants can contact a member of the research team if required. Any participant reporting significant side effects will be reviewed in clinic.

### Concomitant medication

No formal interaction studies have been performed; the clinical protocol excludes patients who are using any other topically administered treatment at the treatment site. As systemic levels are not detectable when applied topically, interaction with other systemic agents is not expected to be an issue. A complete listing of all concomitant medication received during the treatment phase must be recorded in the relevant Case Report Form (CRF).

### Randomisation

A member of the trial team (as outlined in the delegation log) will recheck and confirm eligibility of patients for cohort 2 prior to randomisation. Randomisation will be at the per-patient level, randomising active and placebo treatments to left- or right-sided application. There will be no stratification at randomisation in this early phase trial. The statistics team will be responsible for preparing the allocation lists. Randomisation will be administered centrally by the Newcastle Clinical Trials Unit Internet-accessed secure web-based system, which provides ease of operation with in-built validation/plausibility checks at time of data entry. The Principal Investigator (PI) at site or an individual with delegate authority will access the web-based randomisation system. Patient screening ID and initials will be entered into the web-based system. Patients will be allocated both active and placebo treatments to be applied randomly to left or right tumours (e.g. L = active; R = placebo or L = placebo; R = active, where the terms L and R refer to the patient’s left and right side). Patients allocated to a specific group (e.g. L = active; R = placebo) will remain in this same group when prescriptions are dispensed at baseline and at week 4. Specific training will be given at site initiation.

Four or five tumours matched for size will be selected on each side of the patient. Each tumour will be assigned a number to facilitate assessments, and then each side of the patient will be allocated randomly to receive either active ointment or placebo ointment. Photographs will be taken and the tumours numbered clearly so that the patient can refer to the tumour maps to ensure the correct tumour is being treated from the correct ointment jar. Tumours will be marked with a 2-mm dot using a permanent marker designed for use on the skin, which the patient will renew as necessary. Tumours within the hair will have a small amount of surrounding hair trimmed to allow the patient to locate the tumour by feel, and for volume assessments.

### Blinding

Cohort 2 patients and investigators will be blinded to the treatment allocation. Those responsible for tumour volume measurements, histology assessments and molecular analysis will also be blinded to treatment allocation. The Trial Management Group including the Trial Manager will be blind to the treatment allocation. The trial statistician will provide data to the independent Data Monitoring Committee (DMC) by trial arms A and B (arms will be either 'right active left placebo' or 'left active right placebo'), with an option for unblinding in the closed session (excluding members of the Trial Management Group).

Following the unlikely event of a code break, patients will continue to be followed up if they agree to do so. If the unblinding reveals that the cause was the placebo ointment, patients will be offered the continuation of the trial medication as per the randomisation if they wish. The analysis of the trial will reflect any unblinding as specified in the statistical analysis plan (SAP).

Code breaks will not be routinely performed for all participants who complete trial treatment, but all patients may be informed of their allocations once the trial analysis is complete and the data released to the PI. At the final visit, the blinding will be assessed by asking the participants: Which side was receiving active treatment? Why do you think this? on the patient treatment questionnaire. To avoid bias, the treatment assessor will be asked to record his/her own opinion on a separate CRF and prior to asking the participants their opinions.

### Outcome measures

The primary outcome measure for cohort 1 is the number of severe treated skin site reactions defined as a modified Draize score of 4 or above after 4 weeks of treatment. Additional file 1 describes the modified Draize score, a measure of skin inflammation, in more detail. Secondary outcome measures for cohort 1 are:

- Patient reported quality-of-life tools (EQ5D and DLQI) measured at baseline
- Patient acceptability to trial treatment according to the patient treatment questionnaire measured at the end of treatment visit (week 4)
- Adverse events, graded as mild, moderate or severe, within a planned 4-week treatment period
- Compliance as reported in the patient diary, including reasons for non-compliance, recorded throughout the 4-week treatment period.

The primary outcome measure for cohort 2 is the proportion of tumours responding to treatment by 12 weeks in both actively treated lesions and placebo-treated lesions. Secondary outcome measures for cohort 2 are:

- Change in tumour volume from baseline (pre-randomisation) to 12 weeks assessed by a tumour volume measuring device
- Adverse events, graded as mild, moderate or severe, within a planned 12-week treatment period
- Compliance as reported in the patient diary, including reasons for non-compliance, recorded throughout the 12-week treatment period
- Confirmation of the definition of response (currently according to WHO RECIST)
- Expression of targets of TRK signalling in tumour biopsies as determined by QPCR and immunohistochemistry
- Patient reported quality of life tools (EQ5D and DLQI) measured at baseline
- Assessment of acceptability of trial treatment according to the patient treatment questionnaire measured at the end of treatment visit (week 12)
- Patient reported pain using a trial-specific Patient Pain Assessment Form at 0, 4 and 12 weeks.

In cohort 1 baseline photographs of the selected tumour scheduled for excision will be taken for reference for the eight patients.

In cohort 2 photographs with clearly numbered tumours will be offered to the patients so they can refer to the tumour maps to ensure the correct tumour is being treated from the correct ointment jar and kept at site for reference.

### Tumour volume assessment

All tumours will be assessed for tumour volume using the Life-Viz stereoscopic imaging system by a blinded assessor [12]. This is a validated system that has been shown to be a highly sensitive and reproducible method to assess skin tumour volume assessments. The device will be used to measure the volume of individual tumours. The region of interest, once defined, will be measured as a base area and a raised volume compared to surrounding skin. This measurement of volume and surface area will be taken at 0, 4 and 12 weeks in the trial for each tumour. Tumour-level change in volume at 4 weeks will be reported as mean percentage change (with 95% confidence interval) by randomised treatment group. In order to assess the possibility of differing percentage changes being influenced by initial tumour size, the mean percentage change from baseline to 4 weeks and baseline to 12 weeks will be reported by baseline tumour diameter categories. Mean volume and mean percentage change will be displayed graphically by treatment group and by baseline tumour diameter category. A repeated measures analysis will look at the mean volume over time at baseline, 4 weeks and 12 weeks, accounting for multiple tumours within individual patients and including randomised treatment group as a covariate.

### Tumour biopsies

For cohort 1 patients, at the end of treatment visit (week 4), the routinely excised tumour will undergo routine analysis and some of the sample will be snap-frozen and stored. This sample will be stored with a view to developing a pegcantratinib skin quantitation assay. Participants will have the option to consent to samples potentially being used in future studies relating to the title of this trial.

For cohort 2 patients, at the end of treatment visit (week 12), one tumour that has been treated with active treatment and one tumour that has been treated with placebo will be biopsied to assess histological and immunohistochemical changes and transcriptomic profiling.

### Immunohistochemical analyses and transcriptomic profiling

Tumour samples will be assessed by a pathologist who is blinded to the treatment applied to the specimens. Immunostaining using antibodies against phosphorylated forms of the mitogen-activated protein (MAP) kinase pathway and BCL2 (an antiapoptotic protein) will be assessed to determine if TRK inhibition is leading to anticipated cell death via suppression of the expected downstream targets of TRK. Tissue concentrations of pegcantratinib will also be assessed in a representative portion of samples.

Gene expression profiling of tumour samples to compare 12 placebo- and 12 pegcantratinib-treated samples will be performed as indicated above.

### Data handling and record keeping

The quality and retention of data that are collected as part of this trial are the responsibility of the Principal Investigator, Dr. Neil Rajan. All trial data will be retained in accordance with the latest directive on Good Clinical Practice (GCP) (2005/28/EC) and local policy. Data will be handled, computerised and stored in accordance with the Data Protection Act 1998. Patients will be identified by a unique trial number. Data will be recorded by authorised site staff on electronic Case Report Forms (eCRFs) and stored on a secure validated MACRO database system managed by Newcastle Clinical Trials Unit (NCTU). Caldicott approval will be sought at The Newcastle upon Tyne NHS Foundation Trust site to enable the collection of personal identifiable information for purposes of trial administration (including arranging patient assessments) and maintaining accurate screening records. Data will be extracted from MACRO for statistical analyses in STATA (version 14). STATA data files are stored securely and backed up on a Newcastle University server.

### Trial compliance and withdrawal

Where feasible, trial visits will coincide with routine clinical follow-up to enhance the likelihood of good compliance. Visit windows of +/– 3 days should ensure visit attendance; non-attendance for trial visits will prompt follow-up by telephone. Compliance with trial medication will be assessed by checking the patient diaries. Trial drug accountability will be assessed and documented by local pharmacy. The clinical team will also perform review of any returned trial medication at each trial visit to identify any obvious compliance concerns and address them immediately with the participant.

The trial drug must be discontinued if:

- The participant decides they no longer wish to continue.
- Cessation of the trial drug is recommended by the investigator.
- A participant becomes pregnant.
- Any significant medical condition occurs (in the judgement of the site investigator).
- Localised reactions occur: Any patient reporting treatment site reactions will be assessed in the clinic. A modified Draize score of 4 or above would result in discontinuation of the drug. In the event of such an adverse reaction, blinding may need to be broken to determine if the reaction is related to the application of the trial drug to the treated site. Once the blind is broken, the trial drug will need to be discontinued if it is found to be responsible for the adverse event.
- Systemic reactions occur: Any other reported side effects/adverse events that the patient experiences during the trial will be assessed by the Principal Investigator. If clinically appropriate, the patient will be reviewed in clinic and a decision made regarding discontinuation of the trial drug if it is feasible that this is related to the trial drug.

Participants have the right to completely withdraw from the trial at any time for any reason and without giving a reason. The investigator also has the right to withdraw patients from the trial drug in the event of inter-current illness, adverse events, serious adverse events, suspected unexpected serious adverse reactions, protocol violations, cure, administrative reasons or other reasons. It is understood by all concerned that an excessive rate of withdrawals can render the trial uninterpretable; therefore, unnecessary withdrawal of patients should be avoided. Should a patient decide to withdraw from the trial, all efforts will be made to report the reason for withdrawal as thoroughly as possible. Should a patient withdraw from the trial drug only, efforts will be made to continue to obtain follow-up data, with the permission of the patient.

In both cohorts, additional participants may be recruited, provided withdrawal is not related to safety issues, to preserve the recruitment target based on patients/tumours completing treatment.

### Statistical considerations

#### Statistical definitions and analyses

All statistical analyses will follow a trial-specific predetermined SAP, written and signed off prior to any statistical analysis of the trial data (excepting recruitment) and stored in the Trial Master File.

All cohort 1 patients will contribute to the analysis based on the total number recruited. The primary outcome measure is the number of patients with severe or very severe treated skin site reactions as determined by modified Draize score (see Additional file 1). Patient perception of treatment is a secondary outcome measure as measured by the patient treatment questionnaire. Positive patient perception will be reported descriptively as a proportion of the number of patients recruited. Statistical analysis for all outcome measures will be exploratory based on descriptive data. Continuous measurements (including quality-of-life measures) will be summarised and reported as medians and ranges (inter-quartile ranges). Categorical data (including proportions of patients with positive patient perception and with adverse events) will be summarised and reported as percentages and confidence intervals (95%). Quality-of-life tools (EQ5D, DLQI) will be scored according to scoring manuals, and actual scores will be reported descriptively.

In cohort 2, all tumours will contribute to the analysis based on the total number randomised (intention-to-treat population). A secondary analysis will be reported based on the number of tumours which have been treated for 12 weeks (per protocol population). The primary outcome measure is the number of tumours responding to treatment by 12 weeks reported as a proportion of the number of tumours randomised in the actively and placebo-treated groups separately.

According to WHO RECIST criteria, a reduction in 30% volume would be sufficient to classify a ‘response’ to treatment in oncology. In this trial we will use this criterion as an initial benchmark when determining a response in this patient population. As such, one of the outcomes will be to consider alternative definitions of ‘response’ to treatment in this group of patients. Tumour volume will be measured on 8–10 predetermined identified tumours in each patient prior to randomisation (baseline) and measured again on the same tumours at 4 and 12 weeks. The change in volume for each tumour will be recorded and classified as either showing a response to treatment or not.

As an early phase trial, statistical analysis will be exploratory (not based on hypothesis testing) and will be based on descriptive data presented by treatment group. Continuous measurements (including change in tumour volume from baseline) will be summarised and reported as medians and ranges (inter-quartile ranges). Categorical data (including proportions of tumours with adverse events and outcomes from the Pain Assessment Forms) will be summarised and reported as percentages and confidence intervals (95%). Since multiple tumours are sited in individual patients, a secondary analysis based on a multilevel modelling approach will be undertaken to investigate the relationship between any change in volume and randomised treatment accounting for the multilevel nature of the tumour measurements within patients. Quality-of-life tools (EQ5D, DLQ) will be scored according to manuals and will be reported descriptively.

### Sample size

It is anticipated that there will be low numbers of treatment-related adverse events in cohort 1, and it is therefore both feasible and pragmatic to recruit eight patients. Lack of any unexpected trial-related adverse site reactions is indicated by a modified Draize score of 3 or below, over 4 weeks of treatment. Lack of reactions in at least five of eight treated patients will allow the trial to progress to cohort 2, following recommendation from an independent Data Monitoring Committee.

For cohort 2, it is feasible and pragmatic that 75 tumours could be measured in each treatment arm (150 tumours in total) in a single centre. This is the first exploratory trial; as such, the design parameters are provided as an exemplar of the size of errors that may be anticipated with these patient numbers. Tumours receiving placebo treatment are not expected to respond to treatment, set to be very small at 5% (p0). Using the Fleming-A’Hern early phase methodology [13], any response on the experimental treatment <5% (p0) would not indicate a treatment worthy of further investigation. A level of efficacy of >15% (p1) would indicate a treatment that warranted further investigation. This level of efficacy seems clinically plausible and relevant given that there is no current treatment in use for these patients. Seventy-five tumours recruited in the experimental arm would provide associated error levels of 3.4% type I error (alpha) and 10.8% type II error (beta), acceptable in the early phase setting. The justification to investigate pegcantratinib further is based on observing a minimum number of responses, referred to as the critical number in the experimental arm (as specified in the Fleming-A’Hern design and documented in the Statistical Analysis Plan). The trial will recruit an equal number of placebo-treated tumours to provide an unbiased benchmark.

### Trial monitoring

Monitoring of trial conduct and data collected will be performed by a combination of central review and site monitoring visits to ensure the trial is conducted in accordance with GCP. Trial site monitoring will be undertaken by NCTU. The main areas of focus will include consent, serious adverse events, essential documents in trial files and drug accountability and management.

Site monitoring will include the following tasks:

- ○ All original consent forms will be reviewed as part of the site file. The presence of a copy in the patient hospital notes will be confirmed for 100% of participants.
- ○ All original consent forms will be compared against the trial participant identification lists.
- ○ All reported serious adverse events will be verified against treatment notes/medical records (source data verification).
- ○ The presence of essential documents in the Investigator Site File and other site files will be checked.
- ○ Source data will be verified for primary endpoint data and eligibility data for 100% of participants entered in trial cohort 1 and 50% of participants in trial cohort 2.
- ○ Drug accountability and management will be checked.

Central monitoring will include the following tasks:

- ○ All applications for trial authorisations and submissions of progress/safety reports will be reviewed for accuracy and completeness prior to submission.
- ○ All documentation essential for trial initiation will be reviewed prior to site authorisation.

All monitoring findings will be reported and followed up with the appropriate persons in a timely manner. The trial may be subject to inspection and audit by Newcastle Upon Tyne Hospitals NHS Foundation Trust (NUTH) under their remit as sponsor and other regulatory bodies to ensure adherence to GCP. The investigator(s)/institutions will permit trial-related monitoring, audits, REC review and regulatory inspection(s), providing direct access to source data/documents.

Trial monitoring will be undertaken by three committees with separate roles: (1) Trial Management Group (TMG), (2) Trial Steering Committee (TSC) and (3) Data Monitoring Committee (DMC). The trial will be managed through NCTU. The Principal Investigator, Dr Neil Rajan, will be responsible for the day-to-day trial conduct at site. NCTU will provide day-to-day support for the site and provide training through investigator meetings, site initiation visits and routine monitoring visits. Quality control will be maintained through adherence to NCTU SOPs, the trial protocol, SAP, the principles of GCP, research governance and clinical trial regulations.

A TSC will provide overall supervision of the trial. The TSC will consist of independent chair, further independent clinician, independent consumer representative, funder representative and Trial Management Group. The committee will meet before the start of the trial to make recommendations on the protocol and at least annually thereafter for the duration of the trial. The chair of the TSC and the PI can request meetings more frequently if needed and if also requested by the DMC.

An independent DMC (two independent clinicians and one independent statistician) will undertake independent review and report recommendations to the TSC. The purpose of this committee will be to monitor safety in cohort 1 and recruitment, safety and outcomes in cohort 2. The DMC may request access to unblinded trial data. The DMC will meet at least three times: at the start, following completion of cohort 1 data collection and following completion of cohort 2 data completion.

### Trial reporting

The data will be the property of the Principal Investigator and Co-Investigator(s). Publication will be the responsibility of the Principal Investigator. We plan to publish this trial in peer-reviewed journals and to present data at national and international meetings. Results of the trial will also be reported to the Sponsor and Funder, and will be available on their web site. All manuscripts, abstracts or other modes of presentation will be led by the Trial Management Group and circulated to the Trial Steering Committee and Funder prior to submission. Individuals will not be identified from any trial report. Participants will be informed about their treatment and their contribution to the trial at the end of the trial, including a lay summary of the results.

### SPIRIT

This protocol has been written in accordance with the Standard Protocol Items: Recommendations for Interventional Trials (SPIRIT) guidelines. Please refer to the SPIRIT checklist that was submitted alongside this publication for further details (see Additional file 7).

## Discussion

Patients with rare, germline mutations in *CYLD* develop multiple primary tumours in the skin. We report a trial design which allows within-patient placebo controls, allowing for an unbiased preliminary assessment of TRK inhibition in skin tumours from these patients. This design allows the number of tumours required in each arm to achieve statistical robustness despite the small number of participants typically seen in rare disease. We suggest that this model could be attractive to other researchers involved in rare genetic skin disease.

### Trial status

Cohort 1 of the TRAC trial opened to recruitment on 8 March 2015 and completed recruitment on 23 March 2015. Cohort 2 recruited its first patient on 15 September 2015. Recruitment is due to be complete by July 2016.

## Additional files

Additional file 1: Table showing modified Draize score used to assess for signs of local site reaction in cohort 1. (PDF 84 kb)
Additional file 2: CONSORT diagram for cohort 1: phase 1b of the trial. (PDF 82 kb)
Additional file 3: CONSORT diagram for cohort 2: phase 2a of the trial. (PDF 166 kb)
Additional file 4: Pictorial overview of phase 2a design. (PDF 152 kb)
Additional file 5: SPIRIT figure showing schedule of events in cohort 1 of the trial. (PDF 140 kb)
Additional file 6: SPIRIT figure showing schedule of events in cohort 2 of the trial. (PDF 180 kb)
Additional file 7: SPIRIT 2013 checklist: recommended items to address in a clinical trial protocol and related documents. (DOC 254 kb)

## Abbreviations

AD: Atopic dermatitis
ADR: Adverse drug reaction
AE/AR: Adverse event/adverse reaction
BCC: Basal cell carcinoma
BCL2: B-cell lymphoma 2
CIOMS: Council for International Organisations of Medical Sciences
CTA: Clinical Trial Agreement
*CYLD*: Cylindromatosis gene
CYLD: Cylindromatosis protein
DLQI: Dermatology Life Quality Index
DMC: Data Monitoring Committee
DNA: Deoxyribonucleic acid
DOH: Department of Health
eCRF/CRF: electronic Case Report Form/Case Report Form
ENT: Ear Nose and Throat
EQ5D: European Quality of Life-5 Dimensions
GCP: Good Clinical Practice
HICF: Health Innovation Challenge Fund
ICR: Institute of Cancer Research
IMP: Investigational medicinal product
LSE: Low systemic exposure
MAP: Mitogen-activated protein
MHRA: Medicines and Healthcare Products Regulatory Agency
NCTU: Newcastle Clinical Trials Unit
NuTH: Newcastle upon Tyne Hospitals NHS Foundation Trust
PCI: Packaging Coordinators Inc.
PIS: Patient Information Sheet
PTCH: Patched
QoL: Quality of life
QPCR: Quantitative polymerase chain reaction
REC: Research Ethics Committee
RECIST: Response Evaluation Criteria in Solid Tumours
RNA: Ribonucleic acid
SAE/SAR: Serious adverse event/serious adverse reaction
SOP: Standard Operating Procedure
SUSAR: Suspected unexpected serious adverse reaction
TMG: Trial Management Group
TRK: Tropomyosin receptor kinase
TRKB: Tropomyosin receptor kinase B
TRKC: Tropomyosin receptor kinase C
TSC: Trial Steering Committee
WHO: World Health Organisation
WT: Wellcome Trust
