# Supplementary material for: Tropomyosin Receptor Antagonism in Cylindromatosis (TRAC), an early phase trial of a topical tropomyosin kinase inhibitor as a treatment for inherited CYLD defective skin tumours: study protocol for a randomised controlled trial
Source: Trials. 2017 Mar 7;18:111. doi: 10.1186/s13063-017-1812-z (PMC5341402; doi:10.1186/s13063-017-1812-z)
Supplement: Additional file 1: — Table showing modified Draize score used to assess for signs of local site reaction in cohort 1. (PDF 84 kb) [file 13063_2017_1812_MOESM1_ESM.pdf]

Additional file 1

Score	Grade	Definition
0	Clear	Normal skin surface
1	Almost clear	Just perceptible erythema and just perceptible papulation/infiltration
2	Mild inflammation	Mild erythema and mild papulation/infiltration
3	Moderate inflammation	Moderate erythema and moderate papulation/infiltration
4	Severe inflammation	Severe erythema and severe papulation/infiltration
5	Very severe inflammation	Severe erythema and severe papulation/infiltration with oozing/crusting or ulceration
