# Supplementary figures and images for: Tropomyosin Receptor Antagonism in Cylindromatosis (TRAC), an early phase trial of a topical tropomyosin kinase inhibitor as a treatment for inherited CYLD defective skin tumours: study protocol for a randomised controlled trial
Source: Trials. 2017 Mar 7;18:111. doi: 10.1186/s13063-017-1812-z (PMC5341402; doi:10.1186/s13063-017-1812-z)

## Additional file 2

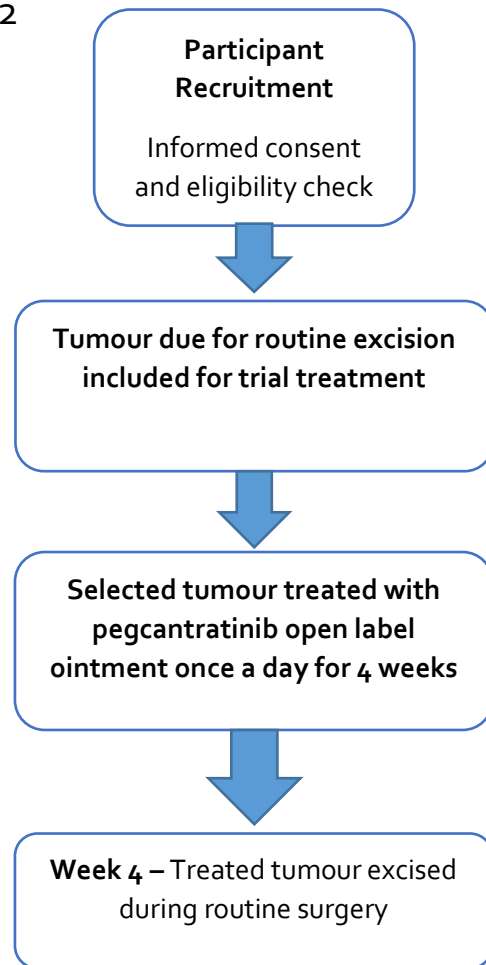

Supplement: Additional file 2: — CONSORT diagram for cohort 1: phase 1b of the trial. (PDF 82 kb) [file 13063_2017_1812_MOESM2_ESM.pdf]

### Additional file 3

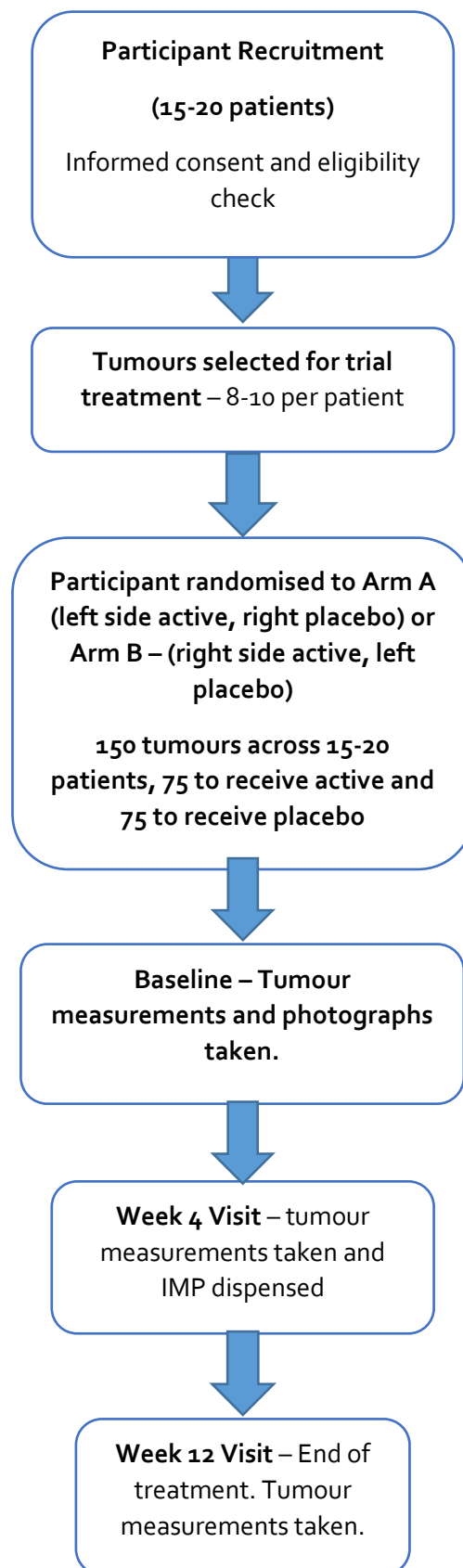

Supplement: Additional file 3: — CONSORT diagram for cohort 2: phase 2a of the trial. (PDF 166 kb) [file 13063_2017_1812_MOESM3_ESM.pdf]
