# Supplementary material for: Tropomyosin Receptor Antagonism in Cylindromatosis (TRAC), an early phase trial of a topical tropomyosin kinase inhibitor as a treatment for inherited CYLD defective skin tumours: study protocol for a randomised controlled trial
Source: Trials. 2017 Mar 7;18:111. doi: 10.1186/s13063-017-1812-z (PMC5341402; doi:10.1186/s13063-017-1812-z)
Supplement: Additional file 4: — Pictorial overview of phase 2a design. (PDF 152 kb) [file 13063_2017_1812_MOESM4_ESM.pdf]

Additional file 4

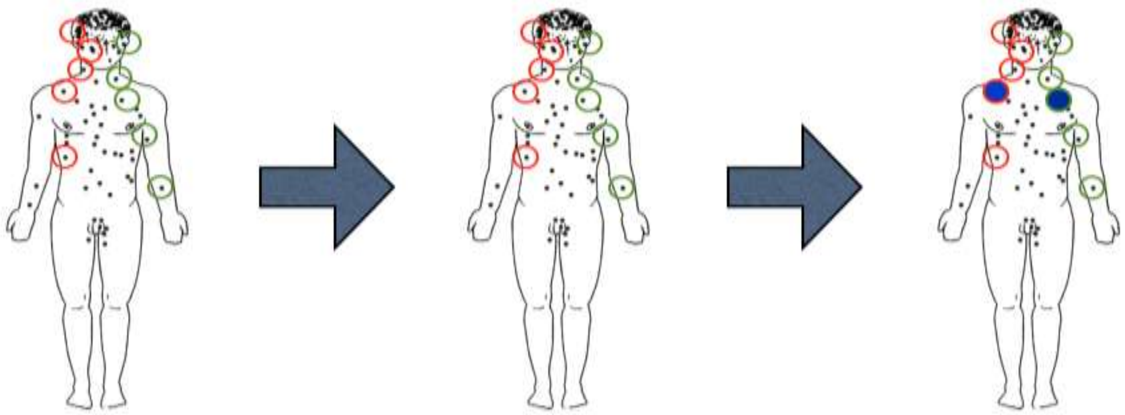

Week 0. 5 matched tumours on left (green circles) and 5 on right (red circles) are selected. Either left or right randomly receive the active drug and the other side placebo.

Week 4.Patient examined and tumour volume assessed.

Week 12. Patient examined and tumour volume assessed, followed by punch biopsy of one tumour from each side (blue circles)
