# Supplementary material for: Tropomyosin Receptor Antagonism in Cylindromatosis (TRAC), an early phase trial of a topical tropomyosin kinase inhibitor as a treatment for inherited CYLD defective skin tumours: study protocol for a randomised controlled trial
Source: Trials. 2017 Mar 7;18:111. doi: 10.1186/s13063-017-1812-z (PMC5341402; doi:10.1186/s13063-017-1812-z)
Supplement: Additional file 5: — SPIRIT figure showing schedule of events in cohort 1 of the trial. (PDF 140 kb) [file 13063_2017_1812_MOESM5_ESM.pdf]

Additional file 5

Time	Visit 1<br>Pre-Screening	Visit 2<br>Baseline visit<br>Confirmation of eligibility			Visit 3a<br>Week 4 EoT	Visit 3b<br>Surgery
	——	Week 0			28 days<br>(+3 days)	28 days<br>(+3 days)
Trial Discussed / PIS given[1]	X					
Informed Consent[2]		X				
Photography				X		
Medical History and demographics			X			
EQ5D			X			
DLQI			X			
Patient treatment questionnaire					X	
Pregnancy Test[3]				X	X	
Trial medication dispensed				X		
Trial medication returned					X	
Patient diary				X	X	
Lesion Excision						X
Adverse events					X	
Concomitant medications			X		X	
CRF completion				X	X	

[1] Patient information sheet can be post to avoid any unnecessary journey.

[2] A minimum of 24hr for review of patient information sheet before patient can sign informed consent form.

[3] Pregnancy test should be urine dipstick and for all women of childbearing potential.
