# Supplementary material for: Tropomyosin Receptor Antagonism in Cylindromatosis (TRAC), an early phase trial of a topical tropomyosin kinase inhibitor as a treatment for inherited CYLD defective skin tumours: study protocol for a randomised controlled trial
Source: Trials. 2017 Mar 7;18:111. doi: 10.1186/s13063-017-1812-z (PMC5341402; doi:10.1186/s13063-017-1812-z)
Supplement: Additional file 6: — SPIRIT figure showing schedule of events in cohort 2 of the trial. (PDF 180 kb) [file 13063_2017_1812_MOESM6_ESM.pdf]

Additional file 6

**Time**	**Visit 1**<br>**Pre-Screening**	**Visit 2**<br>**Baseline visit**<br>**Confirmation of eligibility & Randomisation**			**Telephone call**	**Visit 3**<br>**Week 4 Treatment Visit**	**Telephone call**	**Visit 4**<br>**Week 12 EoT**	**Telephone call**
	**Week -1**	**Week 0**			**Week 2**	**Post Week 4**	**Week 6**	**Post week 12**	**4 weeks post visit 4**
Trial Discussed / PIS given[1]	X								
Informed Consent[2]		X							
Photography				X					
Medical History and demographics			X						
EQ5D[3]			X						
DLQI[3]			X						
Patient treatment questionnaire								X	
Compliance assessment					X		X		
Volume measurement				X		X		X	
Tumour Pain Assessment				X		X		X	
Pregnancy Test[4]			X			X		X	
Blood sample								X	
Trial medication dispensed				X		X			
Trial medication returned						X		X	
Patient Diary				X		X		X	

Lesions biopsied								X	
Adverse events						X		X	X
Concomitant medications			X			X		X	
CRF completion				X		X		X	X

[1] Patient information sheet can be post to avoid any unnecessary journey.

[2] A minimum of 24hr for review of patient information sheet before patient can sign informed consent form.

[3] QoL only to be completed if patient was not part of the cohort 1 trial.

[4]Pregnancy test should be urine dipstick and for all women of childbearing potential.
